# Disaccharide Residues are Required for Native Antifreeze Glycoprotein Activity

**DOI:** 10.1021/acs.biomac.1c00313

**Published:** 2021-05-06

**Authors:** Yuling Sun, Giulia Giubertoni, Huib J. Bakker, Jie Liu, Manfred Wagner, David Y. W. Ng, Arthur L. Devries, Konrad Meister

**Affiliations:** † Max Planck Institute for Polymer Research, 55128 Mainz, Germany; ‡ NWO Institute AMOLF, 1098 XG Amsterdam, The Netherlands; § University of Amsterdam, 1098 XH Amsterdam, The Netherlands; ∥ University of Illinois at Urbana−Champaign, Urbana, Illinois 61801, United States; ⊥ University of Alaska Southeast, Juneau, Alaska 99801, United States

## Abstract

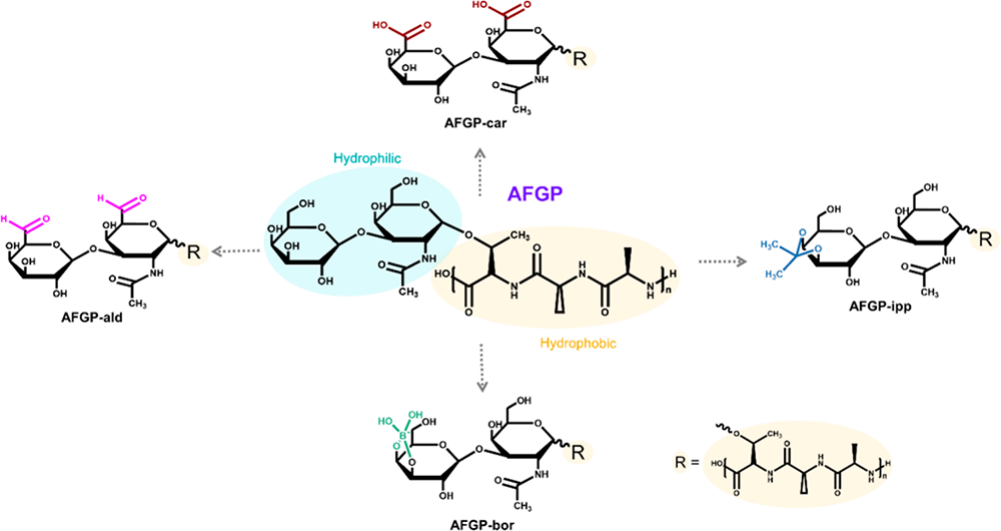

Antifreeze glycoproteins (AFGPs) are able to bind to ice, halt its growth, and are the most potent inhibitors of ice recrystallization known. The structural basis for AFGP’s unique properties remains largely elusive. Here we determined the antifreeze activities of AFGP variants that we constructed by chemically modifying the hydroxyl groups of the disaccharide of natural AFGPs. Using nuclear magnetic resonance, two-dimensional infrared spectroscopy, and circular dichroism, the expected modifications were confirmed as well as their effect on AFGPs solution structure. We find that the presence of all the hydroxyls on the disaccharides is a requirement for the native AFGP hysteresis as well as the maximal inhibition of ice recrystallization. The saccharide hydroxyls are apparently as important as the acetyl group on the galactosamine, the α-linkage between the disaccharide and threonine, and the methyl groups on the threonine and alanine. We conclude that the use of hydrogen-bonding through the hydroxyl groups of the disaccharide and hydrophobic interactions through the polypeptide backbone are equally important in promoting the antifreeze activities observed in the native AFGPs. These important criteria should be considered when designing synthetic mimics.

## Introduction

Antifreeze proteins (AFPs) and antifreeze glycoproteins (AFGPs) are a unique class of macromolecules that inhibit ice growth in the body fluids of organisms, thereby enabling their survival in freezing environments.^1,2^ These natural proteins, as well as synthetic mimics, are of tremendous interest for their use in the cold storage of biological tissues, food, and other water-based materials.^3,4^ All AF(G)Ps have characteristic core activities that include the ability to depress the freezing point in a noncolligative manner without substantially affecting the melting point,^5^ the ability to shape ice crystals into unusual morphologies,^6^ and the ability to inhibit the recrystallization of ice (IRI).^7^ The difference between the freezing point and the melting point is referred to as thermal hysteresis (TH), which is the most characteristic measure for the antifreeze activity of an AF(G)P.^7^ The relative magnitude of each effect varies between different types of antifreeze proteins with the AFGPs exhibiting a moderate hysteresis (1.5 °C) but being by far the most potent of the ice recrystallization inhibitors known.^6^ The molecular details of how the AF(G)Ps achieve their unique antifreeze properties remain largely unknown.^8−11^ It is generally accepted that all AF(G)Ps function by an adsorption-inhibition mechanism in which the proteins recognize and irreversibly bind to specific crystal faces of microscopic ice crystals, thereby preventing macroscopic ice growth.^12^ A long-standing question concerning the mechanism of antifreeze activities of the AFGP is which part of the molecule binds to ice and what forces mediate the interaction. In the case of some fish and insect AFPs of known structure, the ice-binding sites (IBS) have been identified as combinations of flat hydrophobic surfaces and, in some cases, the presence of preordered interfacial water molecules associated with the hydrophobic faces.^13−17^

**Figure 1:**
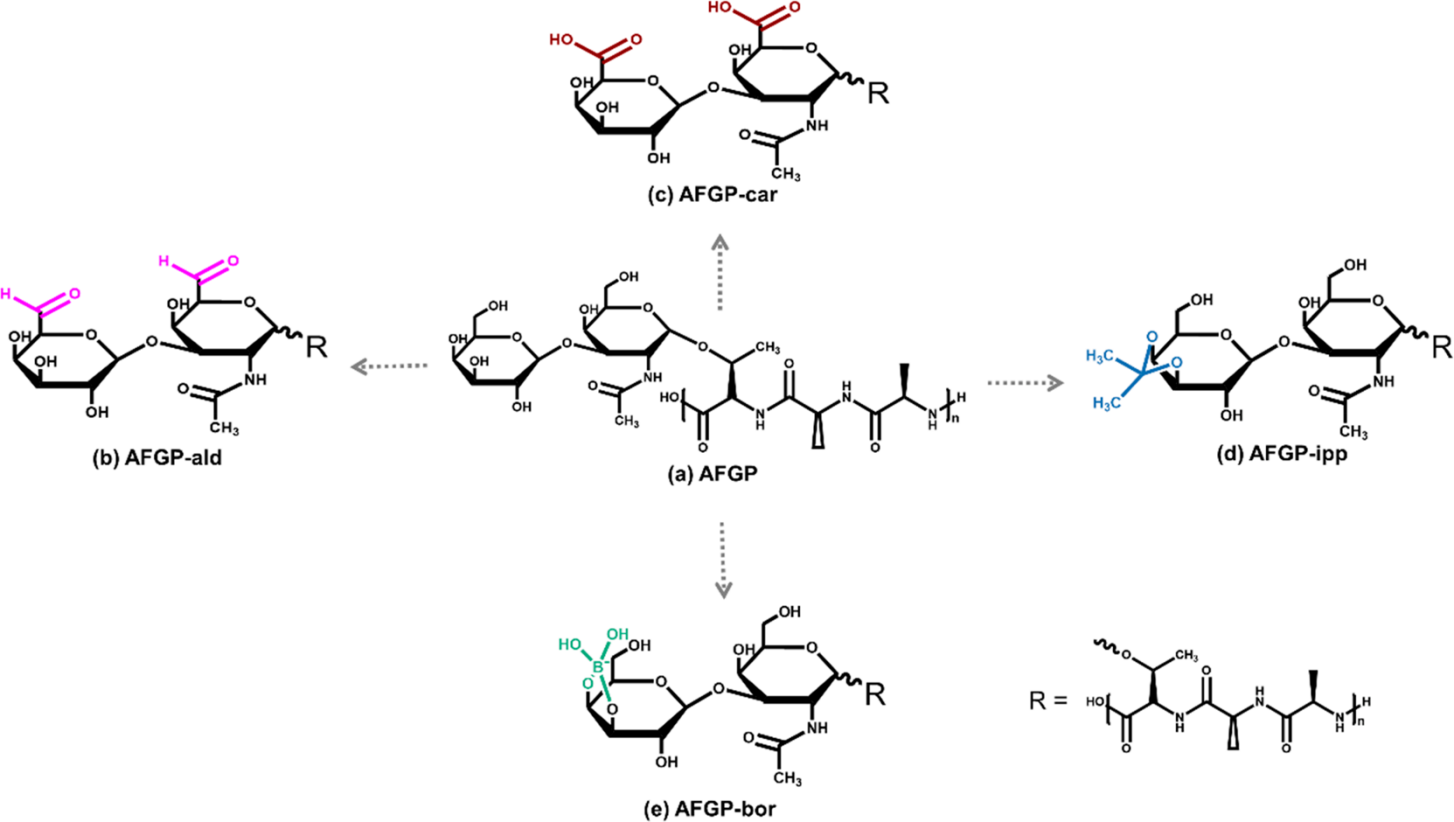
Schematic representation of the natural AFGP and four modified variants. (a) AFGP consists of multiple repetitions of ala-ala-thr units, where the threonine residues are O-glycosylated with the disaccharide, β-d-galactosyl-(1,3)-α-d-acetyl-galactosamine. (b) In the variant AFGP-ald, the C-6 hydroxyl groups of the saccharide were oxidized to aldehyde groups using galactose oxidase. (c) In the variant AFGP-car, the aldehyde groups of AFGP-ald were oxidized to carboxylic acid groups. (d) In variant AFGP-ipp, isopropylidene groups were attached to the carbohydrate. (e) In variant AFGP-bor, borate ions were added and form complexes with the *cis*-hydroxyl groups of the galactose moiety.

AFGPs consist of the repeating tripeptide unit alanine–alanine–threonine (ala-ala-thr), in which the secondary hydroxyl group of the threonine residue is glycosylated with the disaccharide β-d-galactosyl-(1,3)-α-d-acetylgalactosamine (Figure 1a). Nuclear magnetic resonance, two-dimensional infrared spectroscopy, and circular dichroism (NMR, 2D-IR, and CD) investigations suggest that the solution structure of AFGPs is highly flexible and consists of different structural motifs with a prominent polyproline II (PPII) helical content.^10,18,19^ In the PPII conformation, most of the hydrophobic alanines would be clustered on one side with the hydrophilic saccharides on the opposite side. The resulting spacing of some of the AFGP hydroxyls on adjacent disaccharides would be approximately 9 Å, twice the repeat distance of oxygens on the primary prism plane along the *a*-axes.^20^ Likewise, some of the alanines on the hydrophobic side would have a similar spacing.

Recent molecular simulations suggest that AFGPs hydrophobic side binds to ice,^11^ but there is no direct experimental evidence to support this conjecture. Using synthetic analogues Tachahina et al. and others showed that the methyl group of threonine, the acetyl group on carbon 2 of the galactosamine and the α-linkage between the disaccharide and the threonine were essential for the TH activity.^21−23^ Replacing any one of those groups resulted in complete loss of TH activity, indicating the importance of the structural elements located on both the hydrophobic and the hydrophilic sides. Experimental evidence for the importance of the hydroxyl groups of the disaccharide for antifreeze activity remains unclear. Structure-based studies revealed reversible borate inactivation of AFGPs by complexation of borate with the *cis*-hydroxyl groups of the AFGP galactose units.^1,31^

Chemical modifications of natural AFGPs published 50 years ago revealed a complete loss of the TH activity upon hydroxyl group alterations.^24^ However, with these historical modification studies, the freezing points were determined with a freezing point osmometer, which measures the temperature following rapid freezing of a small percentage of the sample. Given the recent findings that the antifreeze activity of AFGPs is freezing rate dependent,^8,25^ the freezing point osmometer would not detect any partial loss of activity associated with the modified hydroxyls. This is because, unlike salts and other small molecules, the AFGP solutions do not form ideal solutions and are sensitive to the rate of freezing. Therefore, we systematically investigated the effect of the hydroxyl groups modifications on antifreeze activities using a Clifton Nanoliter Cryoscope which can control the cooling rate with a temperature resolution of 0.001 °C/min. Using this device, the effect of fast freezing rate is avoided and partial losses of activity of the AFGP variants can be determined because the growth of single ice crystals can be observed as a function of temperature. Independently, the IRI activity was determined, which is another key characteristic associated with antifreeze activity.

## Experimental Section

### Antifreeze Glycoproteins

(AFGP_1–5_, *M*_w_ = 22.1 kDa) were purified from the Antarctic toothfish *Dissostichus mawsoni*, as described previously, and are abbreviated as AFGPs throughout the manuscript.^26^

### Preparation of AFGP-ald

A total of 50 mg of AFGP were dissolved in 2.0 mL of sodium phosphate buffer (0.1 M, pH 6.5). A total of 5 mg of galactose oxidase (Sigma G7400), 1.2 mg of catalase (Sigma C40), and 1 mg of peroxidase (Sigma P825–5KU) were added and incubated at 37 °C for 18 h without a cap so hydrogen peroxide could escape. Then, 5 mg of galactose oxidase, 1.4 mg of catalase, and 1.2 mg of peroxidase were added and incubated for another 8 h. The reaction mixture was then treated with 5% tricholoracetic acid to precipitate the enzymes and the supernatant containing the AFGP-ald was extensively dialyzed in distilled water at 4 °C and then lyophilized. The yield was approximately 45 mg.

### Preparation of AFGP-car

A total of 20 mg of AFGP-ald was dissolved in 3 mL of Milli-Q water. Then, 200 mg of CaCO_3_ was added to maintain the pH during oxidation with bromine water. A total of 15 μL of bromine water was added and gently swirled to disperse the bromine. The solution color was a homogeneous bright yellow. After 2 h of occasional shaking, most of the color had disappeared and another 10 μL of bromine water was added. After 2 h the color of the solution was still yellow and 100 μL of 2% sodium thiosulfate was added to destroy the excess bromine. Approximately 2 mL of 2 M HCl was slowly added to dissolve the CaCO_3_. The oxidized AFGP was dialyzed overnight at 4 °C with two changes of 4 L of deionized water and then lyophilized. The yield of AFGP-car was approximately 20 mg.

### Preparation of AFGP-ipp

A total of 20 mg of dry AFGP was dissolved in 4 mL of *N*,*N*-dimethylformamide (Fisher Chemical). After swirling to dissolve, 2 mL of *N*,*N*-dimethoxypropane (DMP; Sigma 136808) was added, along with a few crystals (∼1 mg) of *p*-toluene-sulfonic acid as a catalyst. The cloudy solution was stirred overnight at room temperature. After 12 h, the solution was clear and another 1 mL of DMP was added and stirred for 6 h. The preparation was lyophilized until nearly dry and then dialyzed in Spectropore 3 (3.5 kDa mol wt cutoff) at 4 °C with three changes of deionized water over 36 h. The yield after lyophilization was approximately 20 mg.

### Preparation of AFGP-ipp-ald

A total of 10 mg of dry AFGP-ald was treated as in the preparation of AFGP-ipp above, except that it was dissolved in 2 mL of DMF and 1 mL of DMP. The remainder of the preparation was identical, as detailed in the AFGP-IPP preparation. The yield was 10 mg.

### Preparation of Borate-AFGP (AFGP-bor)

AFGP was dissolved in a 0.3 M sodium borate, which was prepared by adjusting the pH of 0.3 M boric acid to 9.0 using 4 M NaOH.

### NMR Measurements

NMR measurements were performed in a mixture of 10% D_2_O/90% H_2_O. For the ^1^H NMR and ^13^C NMR experiments (1D and 2D) and diffusion measurements (with water suppression), a 5 mm QXI ^1^H/^13^C/^15^N/^19^F probe equipped with a *z*-gradient on the 700 MHz Bruker AVANCE III system and a 5 mm TXI ^1^H/^13^C/^15^N probe endowed with a *z*-gradient on the 850 MHz Bruker AVANCE III were used. The NMR samples of AFGP, AFGP-ald, AFGP-car, and AFGP-ipp were dissolved in 0.5 mL of 10% D_2_O/90% H_2_O. The AFGP-bor NMR sample was prepared by dissolving AFGP in 0.5 mL of 10% D_2_O/90% H_2_O with 0.3 M borate (pH = 9.0) and subsequent adjustment of the pH to 9.0 using NaOH. Additionally, ^1^H NMR measurements were conducted with water suppression using watergate W5 pulse sequence with gradients and double echo^27^ and referenced with the H_2_O signal at 4.67 ppm (δ(^1^H)). For quantitative ^1^H NMR (700 or 850 MHz) measurements, 64 transients were used with a 9.1 μs long 90° pulse and a 12600 Hz (18 ppm, 700 MHz) spectral width or 17000 Hz (20 ppm, 850 MHz) together with a recycling delay of 8 s. The ^13^C NMR (176 MHz: 90° pulse of 14.5 μs and a spectral width of 240 ppm or 214 MHz: 90° pulse of 12 μs and a spectral width of 260 ppm) measurements were kept with a J-modulated spin–echo for ^13^C-nuclei coupled to ^1^H to determine the number of attached protons (ATP: jmod) with decoupling during acquisition. The temperature was kept at 298.3 K, and the control of the temperature was realized with a VTU (variable temperature unit) and an accuracy of ±0.1 K. The 2D ^1^H,^13^C-HSQC experiments were recorded with 4096 points in f2 and 512 points in f1 dimension (spectral width: 240 ppm, ^1^J_CH_ = 145 Hz) with presaturation during the acquisition and a relaxation delay of 1.5 s.^28−30^

### CD Measurements

CD spectra were recorded at a 1 nm interval from 260 to 180 nm using a Jasco J-1500 spectrometer. CD measurements were performed in a rectangular cell with the optical path of 0.1 cm and at a concentration of 1 mg/mL H_2_O at 22 °C.

### IR/2D-IR Measurements

All linear IR absorption measurements were performed using a Bruker Vertex 80v FTIR spectrometer equipped with a liquid-nitrogen-cooled mercury–cadmium–telluride (MCT) detector. The spectra were recorded under nitrogen atmosphere at a wavelength resolution of 3 cm^–1^. For every spectrum 100 scans were averaged. In all the measurements, a path length of 100 μm was used. The temperature-dependent FTIR measurements were performed using a Peltier-cooled temperature cell (Mid-IR Falcon, Pike technologies). The temperature was ramped from 20 to 5 °C at a rate of 1 °C/min. In all IR and 2D-IR experiments, the proteins were dissolved in heavy water and at a concentration of 2 wt %. The background measurements for pure D_2_O were performed using the same ramping parameters and at the same temperature.

We performed 2D-IR experiments by vibrationally exciting the samples with intense femtosecond mid-infrared pulses centered at 1650 cm^–1^, and probing them with femtosecond pulses centered at 1470 cm^–1^. The details of the setup have been described elsewhere.^31^ The excitation is performed with a mid-infrared pulse pair. This excitation pulse pair induces transient absorption changes that are monitored by a probe pulse that is delayed by a time *T*_w_. After transmission through the sample, the probe pulse is sent into an infrared spectrograph and detected with an infrared mercury–cadmium–telluride (MCT) detector array, thus, yielding the transient absorption spectrum as a function of the probe frequency. The dependence of the transient absorption spectrum on the excitation frequency is determined by measuring transient spectra for many different delay times between the two excitation pulses. By Fourier transformation of these spectra, we obtain the dependence of the transient absorption spectrum on the excitation frequency. By plotting the transient absorption spectrum as a function of the excitation and the probing frequency, we obtain a 2D-IR transient absorption spectrum for each delay time *T*_w_. We measure 2D-IR spectra both for the case that the probe and pump beams have a parallel polarization and the case where they have a perpendicular polarization. All measurements are performed under a N_2_ atmosphere in a standard sample cell with a path length of 100 μm. The temperature of the protein is kept constant by using a Peltier element with an active feedback loop.

### TH Measurements

TH activity was determined at AFGP concentrations of 10 mg/mL in water using a Clifton Nanoliter Osmometer, as described elsewhere.^32^ The hysteresis measurements were performed with a cooling rate of 0.074 °C/min and without annealing. Measurements were preformed multiple times on independent samples.^32^

### IRI Measurements

IRI activity was measured using the splat cooling method^33^ instead of the sucrose method^34,35^ since borate can interact with sucrose, which influences the AFGP–borate binding and makes the results unreliable. AFGP and the modified variants were dissolved in PBS buffer (Dulbecco’s Phosphate-Buffered Saline, 1×, without calcium and magnesium chloride), with a final protein concentration of 2 μg/mL. We chose 2 μg/mL in order to have a maximal IRI activity for the native AFGP.^34,36^

## Results

We study the antifreeze activities (TH, IRI) and the ice habit modifications of the native AFGP, modified disaccharide AFGP variants, and in the presence of borate ions, as shown in Figure 1. The modifications are (I) the AFGP-aldehyde (AFGP-ald) variant obtained by oxidizing the C-6 hydroxyl of the galactose moieties of the natural AFGP isoforms to an aldehyde using galactose oxidase;^24^ (II) the AFGP-carboxyl (AFGP-car) variant obtained by oxidizing the AFGP-ald variant with bromine, and (III) the AFGP-isopropylidene (AFGP-ipp) variant obtained by adding an isopropylidene (IPP) group that replaced the hydroxyls of C-3 and C-4 of the galactose.^24^ (IV) Although technically not a modification, the addition of borate at pH 9.0 results in the formation of a complex with the *cis*-hydroxyls of C-3 and C-4 of the galactose and eliminates most of the antifreeze activity (AFGP-bor).^1,8,37^

**Figure 2:**
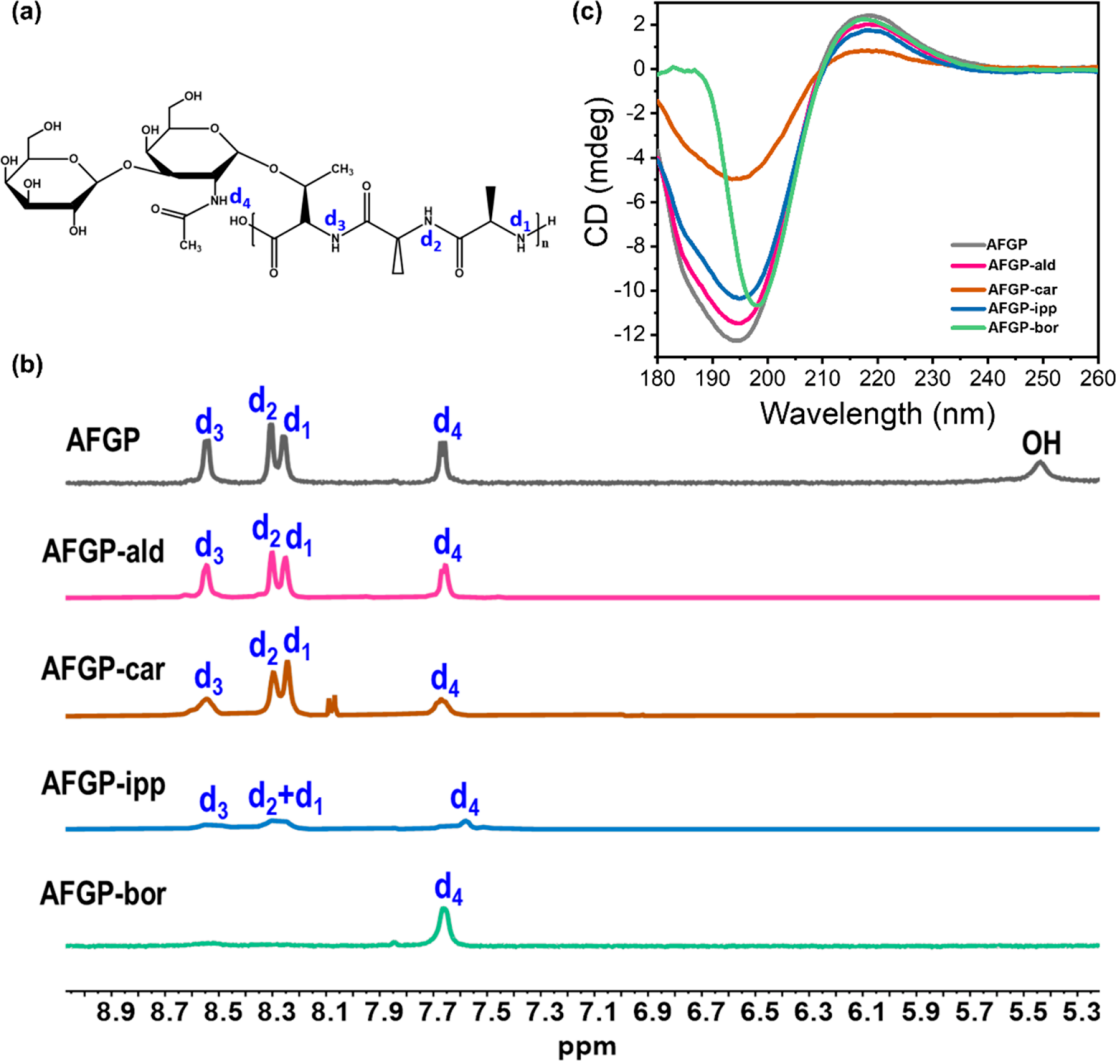
^1^H NMR and CD spectra of AFGP and four modified variants. (a) Chemical structure of AFGP with the assigned amide protons. (b) ^1^H NMR spectra provide information on the hydroxyl groups at ∼5.4 ppm, and the different amides of AFGP and the variants. NMR measurements were performed in a mixture of 10% D_2_O/90% H_2_O and at 700 MHz. (c) CD spectra were measured in water (0.1 mg/mL) and give information on the overall conformation of AFGP and the variants.

In the native AFGP, the hydroxyl groups of the disaccharide moieties show a NMR signal at ∼5.4 ppm, as shown in Figure 2b. Following the modifications, the OH signal at ∼5.4 ppm disappeared in all cases. We confirmed the success of the AFGP-ald modification by observing a new NMR signal at ∼9.2 ppm, which we assigned to the aldehyde proton and an aldehyde specific IR signal at ∼1700 cm^–1^ (SI, Figures S1 and S2). The spectra of AFGP-car also showed a loss of the OH signal, and the presence of a carboxyl group was confirmed by a carboxylic acid specific IR signal at ∼1725 cm^–1^ (SI, Figure S3). The OH signal was also absent in the AFGP-ipp modification, and new signals appeared at ∼1.4 and ∼1.5 ppm, which we assigned to the two new methyl groups (SI, Figure S4). The ipp methyl groups also gave rise to new signals at ∼25.4 and ∼27.2 ppm in the ^13^C NMR spectrum and were confirmed by 2D ^1^H, ^13^C-HSQC ^1^J spectra (SI, Figure S5). Upon addition of borate, the OH signal also disappeared, as expected for a modification of the carbohydrates. We further observe reduced and broadened N–H signals in the presence of 0.3 M borate, which we attribute to changes in the dynamics of the system and which are likely caused by intra- and intermolecular interactions (e.g., borate cross-links between AFGP sugar units). While NMR confirmed the presence of the modification products, quantification was not possible.

Figure 2c shows the CD spectra of AFGP and the variants in the far UV absorption region from 180 to 260 nm. The CD spectrum of the native AFGP shows negative ellipticity centered at ∼195 nm and a positive band at ∼218 nm. The absorption band at ∼195 nm corresponds to the π → π* transition of the carbonyl group and negative ellipticity in this region is associated with random coil and extended PPII helical structures.^38^ The positive *n* → π* transition detected at ∼218 nm is another absorption feature of PPII helices, as demonstrated previously.^38^ In comparison, the CD spectra of the AFGP-ald and AFGP-ipp variants are very similar to that of the native AFGP with no secondary structure changes upon chemical modification of the protein. Notably, the intensity of the CD bands of the AFGP-car variant was reduced at the same concentration, which implies that it has fewer structures that give rise to the CD signals observed in the native form. In the presence of borate, the maximum negative ellipticity showed a slight shift (198 nm) due to the absorption of the B–O bond in the far UV region which effectively shields the CD signal from the protein because the High Tension (HT) Voltage signal in the far UV region exceeds 600 (SI, Figure S6). While the interpretation of the far UV region is interrupted by borate, the positive ellipticity at ∼218 nm corresponding to the PPII helical structures are observable and remain conserved in the presence of borate.

**Figure 3:**
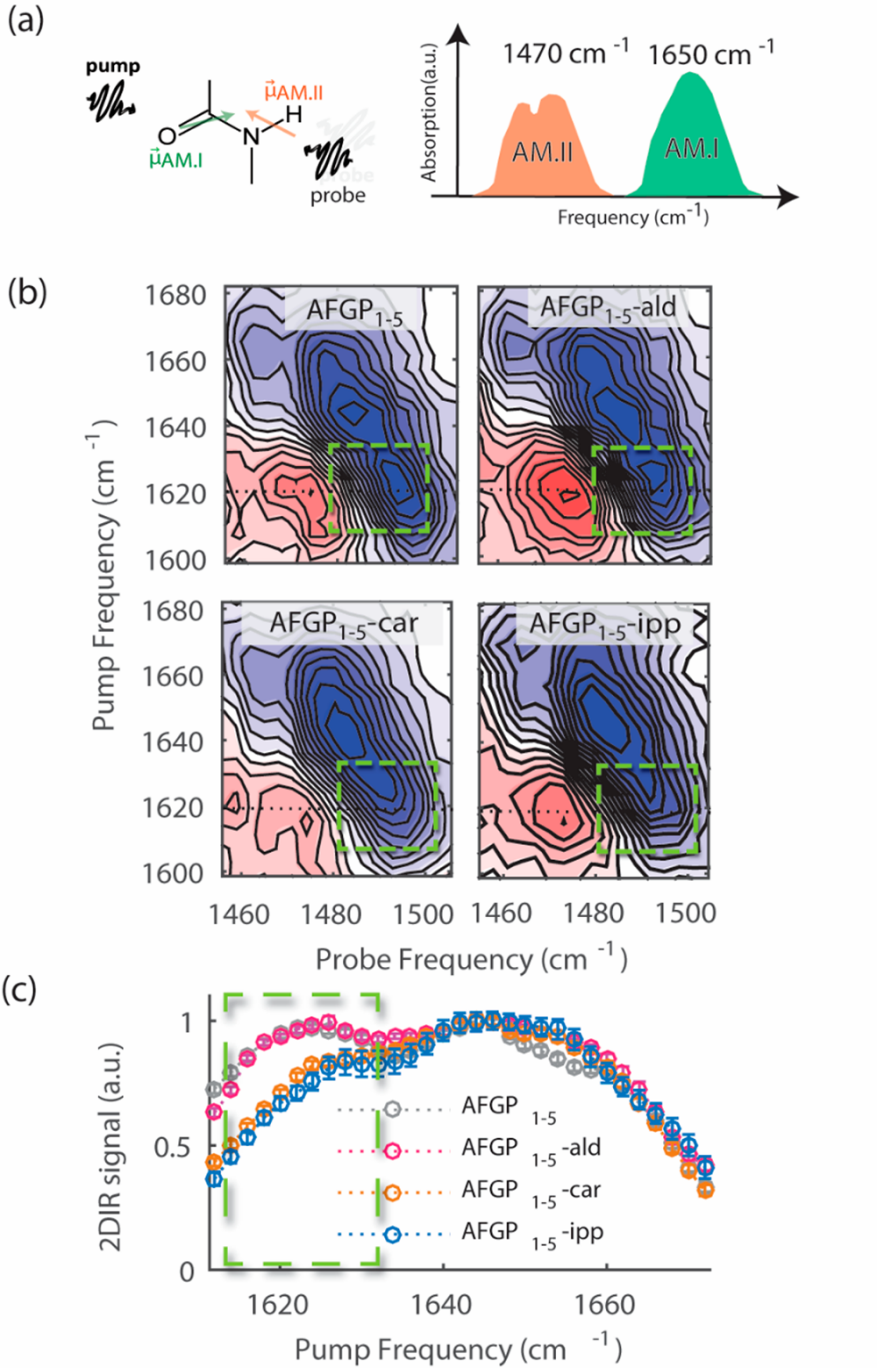
2D-IR experiments of AFGP and the modified variants at 5 °C in heavy water solutions at a concentration of 2 wt %. (a) Schematic representation of 2D-IR measurements, where we excite the amide I (AM.I) vibration around 1650 cm^–1^ with an intense beam (pump), and we probe the amide II (AM.II) vibration at 1470 cm^–1^ with a second beam (probe). Arrows denote transition dipole moments which lie at an angle of ∼65°.^39,40^ (b) Perpendicular 2D-IR spectra of AFGP and modified variants at a concentration of 2 wt %. The waiting time *T*_w_ is 0.5 ps. Blue-colored features represent the bleaching signal (negative-going) and the red-colored features (positive-going) the induced absorption signal. The green rectangles indicate the cross-peak region where we excite the amide I and probe the amide II at the vibrational frequencies characteristic for PPII structures. (c) Normalized antidiagonal slices are taken along the bleaching signal in the spectra shown in (b). The intensity of the 2D-IR signal at the pump frequency of ∼1620 cm^–1^ is lower for AFGP-car and AFGP-ipp.

2D-IR has recently been shown to be suitable to identify different structural motifs and conformations of the AFGPs.^19^ By studying the molecular coupling between the amides I and II vibrations of AFGP, evidence was obtained for the presence of a PPII structure in the AFGP molecule.^19^ The 2D-IR response of the coupled amide I and amide II vibrations was used to study the effect of modifications on the molecular structure of AFGP by excitation of the amide I vibrational modes and by probing the vibrational responses of the amide II vibrational modes. 2D-IR spectra were obtained for the native AFGP and three of the chemically modified variants at 5 °C (Figure 3b). Measurements of the 2D-IR spectrum for AFGP-bor were not possible because of the strong absorption of the antisymmetric B–O stretching vibration of borate in the amide II region.^41^ In Figure 3c we plot the antidiagonal slices along the bleaching component of the cross-peak. The cross-peak signals obtained when exciting around 1620 cm^–1^ has been assigned to PPII structures^19^ and are weaker for AFGP-ipp and AFGP-car than for AFGP-ald and the native AFGP.

To quantify this reduction, we fit the antidiagonal slices using Gaussian-shaped sub bands (SI, Figure S7a). By assuming that the coupling constant between amide I and amide II is similar for the different conformers and different variants, we find that the PPII cross-peak intensity is decreased by 30–40% in AFGP-ipp and AFGP-car with respect to the native AFGP (SI, Figure S7b). This decrease of the relative abundance of the PPII structure is confirmed by fits of the linear IR spectra of the different AFGPs (SI, Figures S8–S11). In these fits, we represent the amide I vibrations of the different conformers with the same Gaussian bands that we found in our previous study of AFGP.^19^ By calculating the area under the single Gaussian-shaped peak, we also obtain the relative fraction of PPII. Assuming a similar cross-section for all the amide I modes, we thus find that solutions of AFGP-bor and AFGP-ald contain a similar content of PPII (27 ± 6% and 27 ± 4%, respectively). AFGP-car and AFGP-ipp contain a significantly lower fraction of PPII (21 ± 4% and 24 ± 5%) shown in Figure S11 (SI), which is in line with the CD results.

We determined the TH activity of the modified AFGPs and compared them to the native AFGP at a concentration of 10 mg/mL using a Nanoliter Cryoscope. When a ∼10 μm disc-shaped seed ice crystal grows in the presence of the native AFGP at a temperature slightly below its melting point, a blunt hexagonal bipyramid (BHBP) forms that morphs into a hexagonal bipyramid (HBP) with a *c*-to-*a* ratio of ∼1.5 as the temperature is slightly lowered. No further growth is observed until a point is reached where the bound AFGPs can no longer constrain crystal growth and usually a single, fine spicule propagates from the tips of the HBP, which appear to thicken slightly, followed by rapid propagation of several more spicules growing parallel to the initial ones (SI, Movie S1). This burst of spicular grow is referred to as the nonequilibrium freezing point. The difference between this burst temperature and the melting point is referred to as thermal hysteresis.

**Figure 4:**
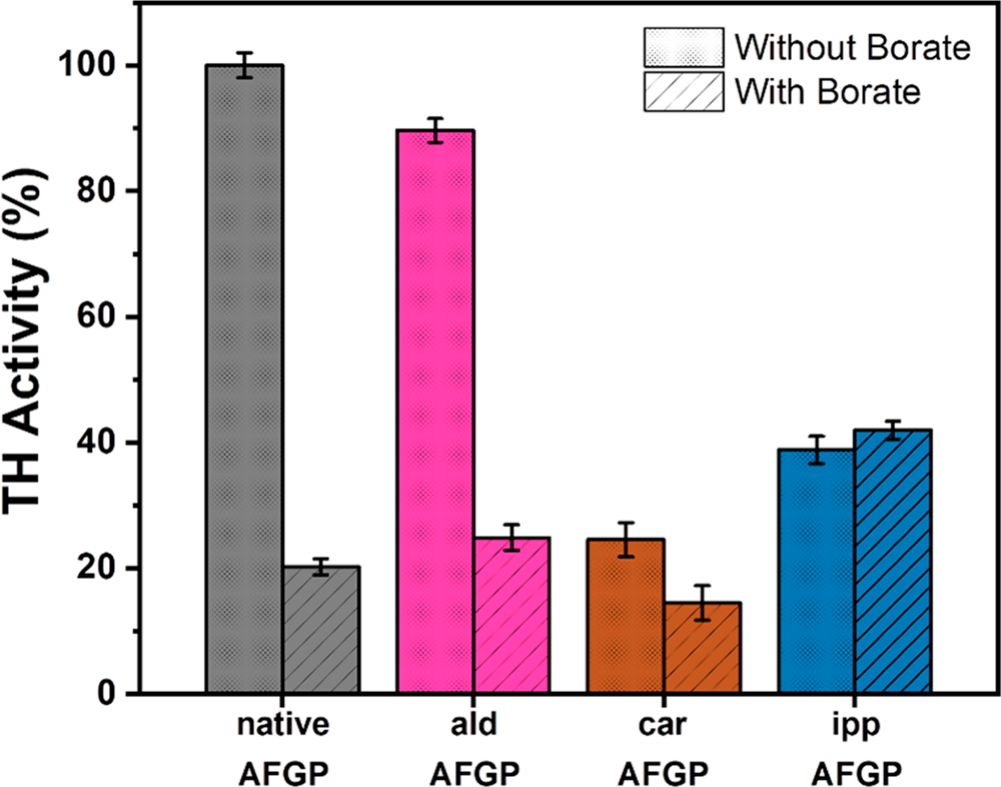
TH activity measurements of AFGP and the modified variants. The TH activity at 10 mg/mL for AFGP-ald, AFGP-car, AFGP-ipp, and AFGP-bor in water are reduced by ∼10, 75, 61, and 80%, respectively. In the presence of borate, the activity of AFGP-ald, AFGP-car, and AFGP-ipp are reduced by ∼75, 86, and 58%. Experiments were performed at least three times, and the error bars represent the standard deviation between the individual measurements.

The AFGP-ald hysteresis was ∼90% of the native AFGP (0.7 °C), as shown in Figure 4. AFGP-ald’s ice growth in the hysteresis gap was similar to that of the native AFGP (SI, Movie S2). The TH activities of the AFGP-car and the AFGP-ipp were about ∼25% and ∼39% of the native AFGP. In the presence of these variants, the seed crystals grew from a disc into a BHBP and quickly morphed into a HBP just below the melting point and continued to grow along the *c*-axis as the temperature was lowered with the tips extending toward the interface but without an increase in the width of the original HBP (SI, Movies S3 and S4). The tip growth stopped upon reaching the interface and *a*-axes growth ensued producing a thick single crystal spear without further cooling. Overall, the ice growth pattern in the presence of variants AFGP-car and AFGP-ipp were similar to that of the small AFGP isoform 8 (SI, Movie S5).

**Figure 5:**
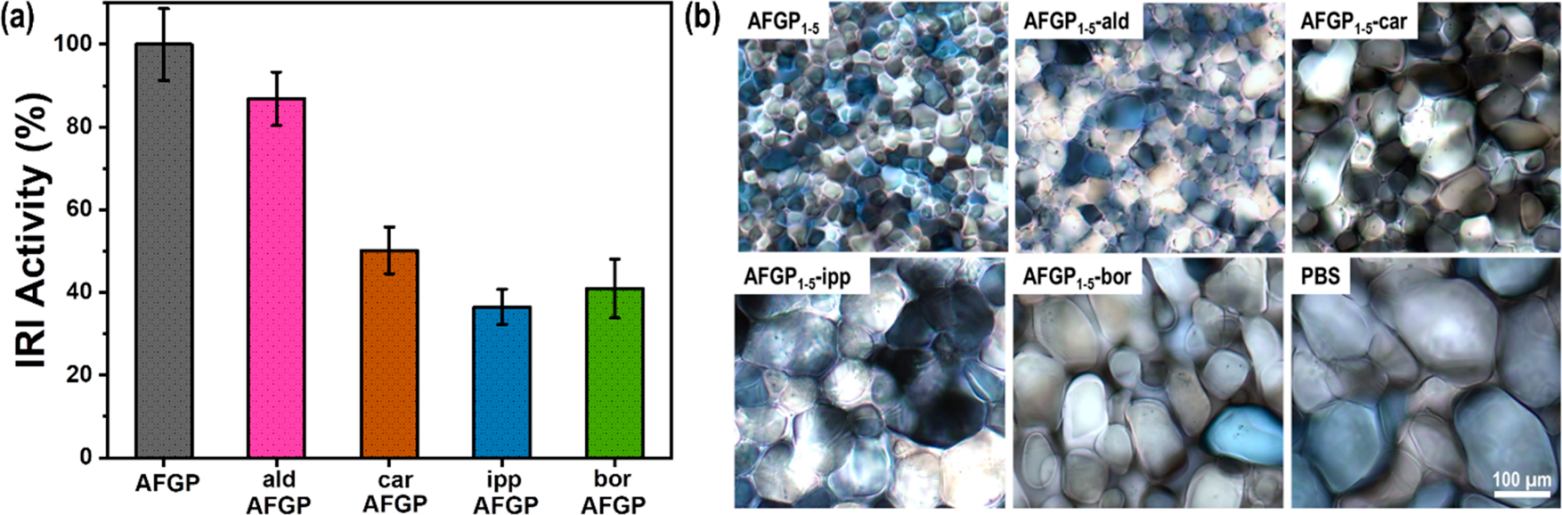
IRI activity measurements of AFGP and the modified variants. (a) The IRI activity of AFGP-ald, AFGP-car, AFGP-ipp, and AFGP-bor at 2 μg/mL are reduced by ∼13, 50, 63, and 59%, respectively. (b) Cryomicroscopic image showing attenuation of ice crystal growth by the addition of native AFGP and modified AFGPs annealed at −6 °C for 30 min. Experiments were performed at least three times, and the error bars represent the standard deviation between the individual measurements.

The addition of borate ions to the native AFGP causes a complex formation with the *cis*-hydroxyls of C-3 and C-4 of the galactose that largely reduces hysteresis activity.^8^ We determined the hysteresis of all variants in the presence of borate, as shown in Figure 4. The TH activity of the AFGP-bor determined here was 20% of that of the native AFGP, which agrees with previous studies.^8,11^ In the case of AFGP-ald and AFGP-car, we observed that the activity in the presence of borate decreases further to 25% and 14% of the native AFGP activity. For AFGP-ipp it was found that there is no further decrease in TH activity, indicating that borate has no effect on the unmodified OH groups, presumably because it only forms a complex with the OH groups of C-3 and C-4 of the galactose which were unavailable in the ipp variants. To further investigate this hypothesis, we prepared an additional variant, AFGP-isopropylidene-aldehyde (AFGP-ipp-ald), obtained by adding an IPP group to the AFGP-ald variant. Upon borate addition, the TH activity of AFGP-ipp-ald did not decrease and remained at ∼40% (SI, Figure S12). The IRI activity which is the second main antifreeze activity was also significantly affected by the hydroxyl modification as shown in Figure 5. At 2 μg/mL AFGP in phosphate buffered saline, the ice grain boundary migration, in which large ice crystals increase in size and small crystals disappear (i.e., Ostwald ripening) was clearly prevented. The IRI activity of AFGP-ald was reduced by 13% while AFGP-car and AFGP-ipp were about 50% and 63% of that of the native AFGP. Likewise, the IRI activity of AFGP in the presence of borate was reduced by 59%.

## Discussion

The specific structural elements necessary for TH activity and the relation between IRI and TH activities have remained unclear, and thus key requirements for the de novo design of efficient antifreeze mimics are not apparent. In fact, up to now, no synthetic analogue shows TH activity, and even the most active synthetic IRI compounds are several orders of magnitude less active than their natural counterparts.^6^ For AFGP activity, the importance of the α-linkage between the disaccharide and the threonine, the presence of the N-acetyl group, and the methyl groups of alanines and threonines have been noted.^23^ Historical hydroxyl modification studies using a freezing point osmometer further suggested that the presence and positions of the hydroxyl groups on the disaccharide are important.^42,43^ Among 12 modifications, 10 completely abolished antifreeze activity with the exception being the galactose aldehyde^24^ and its iodine-oxidized derivative.^43^ Freezing point osmometers have, however, been shown to inadequately determine TH activities due to the fast freezing process.^25^ When the TH activity was assessed by visually determining the ice growth behavior of a small ice crystal, all the hydroxyl modifications reported here revealed partial rather than complete loss of activity. The AFGP-ipp, AFGP-car, and AFGP-bor all lost 60–80% of their TH activity, while the AFGP-ald lost only 10% of its activity. The modifications further altered the growth morphology in the hysteresis gap compared to the native AFGP and reduced the IRI activities to a similar extent as the TH activities.

Interestingly, the ice growth morphologies of the variants with the lowest TH activities (AFGP-ipp AFGP-car, and AFGP-bor) were more similar to those of the small native AFGP_7–8_ isoforms. Given the similarity, we suggest that the modifications decrease the ice binding affinity compared to the native AFGP which leads to a reduction in TH, as recently shown for AFGP activity in the presence of borate.^8^ Our results agree with previous findings that borate is known to bind reversibly to diol-containing compounds,^44^ and that borate can inactivate AFGPs by complexing the *cis*-hydroxyl groups of the β-d-galactopyranosyl groups.^1,42^ We speculate that as the HBP extends in the hysteresis gap the advancing ice fronts on the pyramidal planes push the weakly adsorbed AFGPs off the plane allowing growth of the pyramidal planes as the tips extend.

Although some of the modified AFGPs lose up to 80% of their antifreeze activity it remains unclear what is responsible for the residual 20% activity. It is possible that the unmodified hydroxyl groups C-2 and C-6 on the galactose and C-4 and C-6 on the acetyl-galactosamine still promote some weak ice binding. Alternatively, it is possible that ice binding occurs through the alanine methyl groups in those parts of the AFGPs that are still in the PPII-helical conformation.

PPII helices separate the hydrophilic and hydrophobic part of the AFGP molecules and it has been suggested that this amphipathcity plays a role in ice binding with the hydrophilic face on one side of the helix and the hydrophobic methyl groups of the alanines on the other side.^20^ A question that remains is whether the AFGPs bind to ice via the hydrophilic saccharides or the hydrophobic methyl groups of the alanines. What is clear from studies using synthetic short AFGPs is that both the specific backbone and the disaccharide are required to realize native antifreeze activity.^23^ The data presented here also implicates the importance of specific hydroxyl groups on the saccharide for optimal antifreeze activity. One of the difficulties in interpreting the effect of the hydroxyl modification on antifreeze activity is whether the loss of activity is only due to the alteration of the hydroxyl groups or whether it causes a change in the overall structure and a reduction of the PPII helical content. The CD and 2D-IR structural analysis showed that AFGP-ald and AFGP-bor showed little difference in their spectra compared to the native and had a similar content of PPII helix. In contrast, AFGP-car and AFGP-ipp contained a reduced PPII helical content. The CD spectrum of the native AFGP is thought to arise from that part of the molecule that is in the form of a PPII helix.^38^ Although there is a consensus that the CD spectrum represents that of a PPII helix, it is only a small percentage of the molecular structure that is in this conformation at least in solution.^23^ Most of the remainder of the molecule is thought to be unstructured and, if so, how this part of the molecule interacts with ice remains unknown.

According to previous reports,^14,16^ the ice-binding sites of AFPs can influence the arrangement of water molecules around them. For the AFGPs we did not find direct evidence for preordered water layers, which is in agreement with previous simulation studies.^10,45^ Unlike the structurally rigid AFPs for which ice-like or anchored clathrate water domains have been reported (e.g., AFP type III),^16^ the AFGPs are highly flexible in solution and have repetitive carbohydrate residues. AFGPs might already by themselves provide a good surface similarity to ice and thus do not require additional ordered water layers to obtain a high ice surface affinity. We speculate that while AF(G)P-induced enhanced interfacial water ordering likely constitutes an important part of all AF(G)Ps working mechanism, the extent and properties of the preordered water domains differ between different AF(G)P classes.

## Conclusions

In conclusion, it is clear that the incremental removal or alterations of the saccharide hydroxyl groups diminishes the TH and IRI activities in a generally similar way. Alteration of the C-6 hydroxyl to an aldehyde causes a slight loss of activity with a small change in growth habit, while conversion of the aldehyde to a carboxyl group causes slightly more loss of activity and more change in the growth habit in the hysteresis gap. Alteration of two or more of the hydroxyl groups diminishes the activity to 30% of that of the native as shown by additional experiments in borate buffer. These findings attest to the importance of hydroxyl groups for native activity. Considering the evidence from previous synthetic antifreeze analogues and modifications of the saccharide hydroxyl groups on antifreeze activity, it appears that the question as to whether it is the hydrophilic or hydrophobic side that binds to ice cannot be answered with the available evidence. What is abundantly clear from the present study and others is that there is a delicate balance between the saccharides (hydrophilic) and polypeptide (hydrophobic) moieties associated with the native antifreeze activity, and we think that an amphipathic balance is a general requirement for all classes of antifreeze proteins. We further speculate that implementing an amphipathic balance in the design of novel AFGP mimics will have a transformative impact on their capabilities and subsequent use in cryopreservation and anti-icing applications.

## Supporting Information Available

NMR spectra of different AFGP variants (AFGP-ald, AFGP-car, AFGP-ipp, AFGP-ipp-ald, and AFGP-bor), measurement details (CD, IR/2D-IR, TH measurement, IRI measurement), linear IR spectra of AFGP, AFGP-ald, and AFGP-car, HT(V) spectrum of AFGP and AFGP-bor variant, antidiagonal slices and normalized areas of the five 2D-IR bands for AFGP and variants, linear IR spectra and fits for AFGP and variant solutions, linear IR spectra and detailed PPII absorption of AFGP and variant solutions, 2D-IR spectrum of AFGP-car and linear spectrum of glucuronic acid, relative areas of the PPII band for AFGP and variant solutions, TH activity of AFGP and AFGP-ipp-ald, and legends of Movies S1–S5 (PDF)
Movie S1 (AVI)
Movie S2 (AVI)
Movie S3 (AVI)
Movie S4 (AVI)
Movie S5 (AVI)

## Supplementary Material

bm1c00313_si_001.pdf

bm1c00313_si_003.avi

bm1c00313_si_004.avi

bm1c00313_si_005.avi

bm1c00313_si_006.avi

bm1c00313_si_007.avi
