# Supplementary material for: Disaccharide Residues are Required for Native Antifreeze Glycoprotein Activity
Source: Biomacromolecules. 2021 May 6;22(6):2595–603. doi: 10.1021/acs.biomac.1c00313 (PMC8207503; doi:10.1021/acs.biomac.1c00313)
Supplement: Supplementary file 1 — bm1c00313_si_001.pdf [file bm1c00313_si_001.pdf]

Supporting Information

Disaccharide Residues are Required for Native Antifreeze Glycoprotein Activity

*Yuling Sun[1], Giulia Giubertoni[2,3], Huib J. Bakker[2], Jie Liu[1], Manfred Wagner[1], David Y. W. Ng[1], Arthur L. Devries[4], Konrad Meister[1,5]**

[1]Max Planck Institute for Polymer Research, 55128 Mainz, Germany

[2]NWO Institute AMOLF, 1098 XG Amsterdam, The Netherlands

[3]University of Amsterdam, 1098 XH Amsterdam, The Netherlands

[4]University of Illinois at Urbana–Champaign, Urbana, IL, 61801, USA

[5]University of Alaska Southeast, Juneau, AK, 99801, USA

Corresponding Authors

K. Meister − Max Planck Institute for Polymer Research, 55128 Mainz, Germany; University of Alaska Southeast, Juneau, Alaska 99801, United States; orcid.org/0000-0002-6853-6325; Email: meisterk@mpip-mainz.mpg.de

This file includes:

1) Materials and Methods
2) Figures S1 to S12
3) Legends for Movies S1 to S5

Materials and Methods

Antifreeze glycoproteins ($AFGP_{1\text{-}5}$, M_w = 22.1 kDa) were purified from the Antarctic toothfish *Dissostichus mawsoni* as described previously and are abbreviated as AFGPs throughout the manuscript[1].

Preparation of AFGP-ald. 50 mg of AFGP were dissolved in 2.0 mL sodium phosphate buffer (0.1 M, pH 6.5). 5 mg galactose oxidase (Sigma G7400), 1.2 mg catalase (Sigma C40), and 1 mg peroxidase (Sigma P825-5KU) were added and incubated at 37 °C for 18 hours without a cap so hydrogen peroxide could escape. Then, 5 mg galactose oxidase, 1.4 mg catalase and 1.2 mg peroxidase were added and incubated for another 8 hours. The reaction mixture was then treated with 5% tricholoracetic acid to precipitate the enzymes and the supernatant containing the AFGP-ald was extensively dialyzed in distilled water at 4 °C and then lyophilized. The yield was approximately 45 mg.

Preparation of AFGP-car. 20 mg of AFGP-ald was dissolved in 3 mL milliQ water. Then, 200 mg $CaCO_3$ was added to maintain the pH during oxidation with bromine water. 15 µL bromine water was added and gently swirled to disperse the bromine. The solution color was a homogenous bright yellow. After 2 hours of occasional shaking, most of the color had disappeared and another 10 µL of bromine water was added. After 2 hours the color of the solution was still yellow and 100 µL of 2% sodium thiosulfate was added to destroy the excess bromine. Approximately 2 mL of 2 M HCl was slowly added to dissolve the $CaCO_3$. The oxidized AFGP was dialyzed overnight at 4 °C with two changes of 4 L of deionized water and then lyophilized. The yield of AFGP-car was approximately 20 mg.

Preparation of AFGP-ipp. 20 mg of dry AFGP was dissolved in 4 mL of N,N-dimethyl formamide (Fisher Chemical). After swirling to dissolve, 2 mL of N,N-dimethoxypropane (DMP) (Sigma 136808) was added along with a few crystals (~1 mg) of p-toluene-sulfonic acid as a catalyst. The cloudy solution was stirred over night at room temperature. After 12 hours the solution was clear and another 1 mL of DMP was added and stirred for 6 hours. The preparation was lyophilized until nearly dry and then dialyzed in Spectropore 3 (3.5 kDa mol wt cutoff,) at 4 °C with 3 changes of deionized water over 36 hours. The yield after lyophilization was approximately 20 mg.

Preparation of AFGP-ipp-ald. 10 mg of dry AFGP-ald was treated as in the preparation of AFGP-ipp above, except that it was dissolved in 2 ml of DMF and 1 ml of DMP. The remainder of the preparation was identical as detailed in the AFGP-IPP preparation. The yield was 10 mg.

Preparation of Borate-AFGP (AFGP-bor). AFGP was dissolved in a 0.3 M sodium borate which was prepared by adjusting the pH of 0.3M boric acid to 9.0 using 4 M NaOH.

NMR measurements. NMR measurements were performed in a mixture of 10% D_2O/90% H_2O. For the ^{1}H-NMR and ^{13}C-NMR experiments (1D and 2D) and diffusion measurements (with water suppression), a 5 mm QXI ^{1}H/^{13}C/^{15}N/^{19}F probe equipped with a z-gradient on the 700 MHz Bruker AVANCE III system and a 5 mm TXI ^{1}H/^{13}C/^{15}N probe endowed with a z-gradient on the 850 MHz Bruker AVANCE III were used. The NMR samples of AFGP, AFGP-ald, AFGP-car, and AFGP-ipp were dissolved in 0.5 mL 10% D_2O/90% H_2O. The AFGP-bor NMR sample was prepared by dissolving AFGP in 0.5 mL 10% D_2O/90% H_2O with 0.3 M borate (pH=9.0) and subsequent adjustment of the pH to 9.0 using NaOH. Additionally ^{1}H-NMR measurements were conducted with water suppression using watergate W5 pulse sequence with gradients and double echo[2] and referenced with the H_2O signal at 4.67 ppm (δ(^{1}H)). For a quantitative ^{1}H-NMR (700 MHz or 850 MHz) measurements 64 transients were used with a 9.1 µs long 90° pulse and a 12600 Hz (18 ppm, 700 MHz) spectral width or 17000 Hz (20 ppm, 850 MHz) together with a recycling delay of 8 s. The ^{13}C-NMR (176 MHz: 90° pulse of 14.5 µs and a spectral width of 240 ppm or 214 MHz: 90° pulse of 12 µs and a spectral width of 260 ppm) measurements were kept with a J-modulated spin-echo for ^{13}C-nuclei coupled to ^{1}H to determine number of attached protons (ATP: jmod) with decoupling during acquisition. The temperature was kept at 298.3 K and the control of the temperature was realized with a VTU (variable temperature unit) and an accuracy of +/- 0.1 K. The 2D ^{1}H,^{13}C-HSQC experiments were recorded with 4096 points in f2 and 512 points in f1 dimension (spectral width: 240 ppm, $^1J_{CH}$ = 145 Hz) with presaturation during the acquisition and a relaxation delay of 1.5 s[3-5].

CD measurements. CD spectra were recorded at a 1 nm interval from 260 nm to 180 nm using a Jasco J-1500 spectrometer. CD measurements were performed in a rectangular cell with the optical path of 0.1 cm and at a concentration of 1 mg/mL H_2O at 22 ^{o}C.

IR/2D-IR measurements. All linear IR absorption measurements were performed using a Bruker Vertex 80v FTIR spectrometer equipped with a liquid-nitrogen-cooled-mercury-cadmium-telluride (MCT) detector.

The spectra were recorded under nitrogen atmosphere at a wavelength resolution of 3 cm^{-1}. For every spectrum 100 scans were averaged. In all the measurements, a path length of 100 μm was used. The temperature-dependent FTIR measurements were performed using a Peltier-cooled temperature cell (Mid-IR Falcon, Pike technologies). The temperature was ramped from 20 to 5 ˚C at a rate of 1 ˚C/min. In all IR and 2D-IR experiments, the proteins were dissolved in heavy water and at a concentration of 2 wt%. The background measurements for pure D_2O were performed using the same ramping parameters and at the same temperature.

We performed 2D-IR experiments by vibrationally exciting the samples with intense femtosecond mid-infrared pulses centred at 1650 cm^{-1}, and probing them with femtosecond pulses centred at 1470 cm^{-1}. The details of the setup have been described elsewhere[6]. The excitation is performed with a mid-infrared pulse pair. This excitation pulse pair induces transient absorption changes that are monitored by a probe pulse that is delayed by a time *Tw*. After transmission through the sample, the probe pulse is sent into an infrared spectrograph and detected with an infrared mercury-cadmium-telluride (MCT) detector array, thus yielding the transient absorption spectrum as a function of the probe frequency. The dependence of the transient absorption spectrum on the excitation frequency is determined by measuring transient spectra for many different delay times between the two excitation pulses. By Fourier transformation of these spectra, we obtain the dependence of the transient absorption spectrum on the excitation frequency. By plotting the transient absorption spectrum as a function of the excitation and the probing frequency, we obtain a 2D-IR transient absorption spectrum for each delay time *Tw*. We measure 2D-IR spectra both for the case that the probe and pump beams have a parallel polarization, and the case where they have a perpendicular polarization. All measurements are performed under N_2 atmosphere in a standard sample cell with a path length of 100 μm. The temperature of the protein is kept constant by using a Peltier element with an active feedback loop.

TH measurements. TH activity was determined at AFGP concentrations of 10 mg/mL in water using a Clifton Nanoliter Osmometer as described elsewhere[7]. The hysteresis measurements were performed with a cooling rate of 0.074 ^{o}C/min and without annealing. Measurements were preformed multiple times on independent samples[7].

IRI measurements. IRI activity was measured using the splat cooling method[8] instead of sucrose method[9-10] since borate can interact with sucrose which influences the AFGP-borate binding and makes the results unreliable. AFGP and the modified variants were dissolved in PBS buffer (Dulbecco's Phosphate-Buffered Saline, 1×, without calcium and magnesium) with a final protein concentration of 2 μg/mL. We chose 2 μg/mL in order to have a maximal IRI activity for the native AFGP[9, 11].

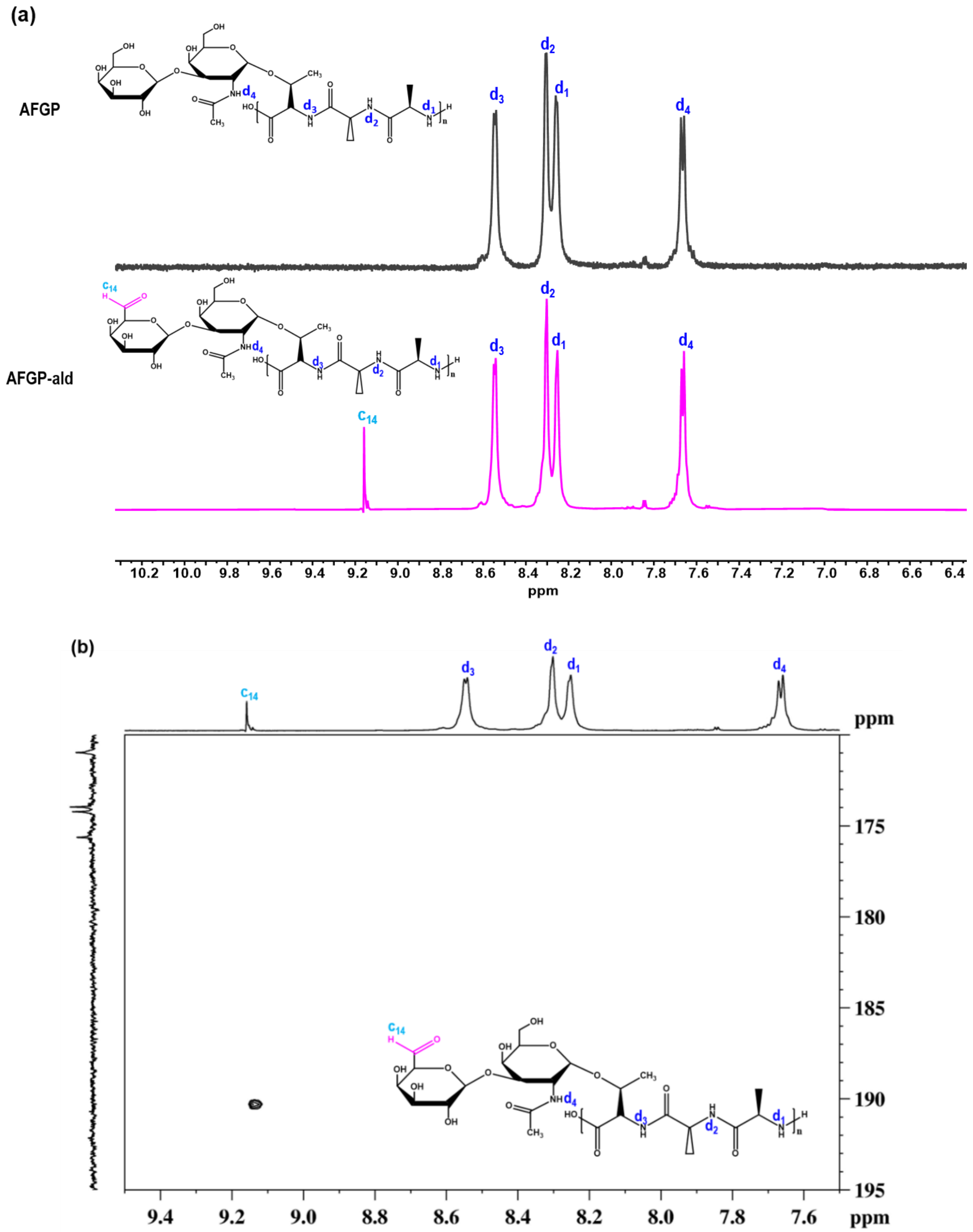

Figure S1. ^{1}H-NMR and 2D ^{1}H, ^{13}C-HSQC ^{1}J spectra of AFGP and AFGP-ald in the region from 7.5 to 9.4 ppm. (a) ^{1}H -NMR spectra shows the new signal at ~9.2 ppm (peak c_{14}) is assigned to proton of the aldehyde group. (b) 2D ^{1}H,^{13}C-HSQC ^{1}J spectra proves peak c_{14} is from AFGP-ald. In the 2D HSQC experiment, which is sensitive for ^{1}J coupling shows clearly a correlation between the aldehyde proton at 9.2 ppm and the carbonyl carbon atom at 190.2 ppm.

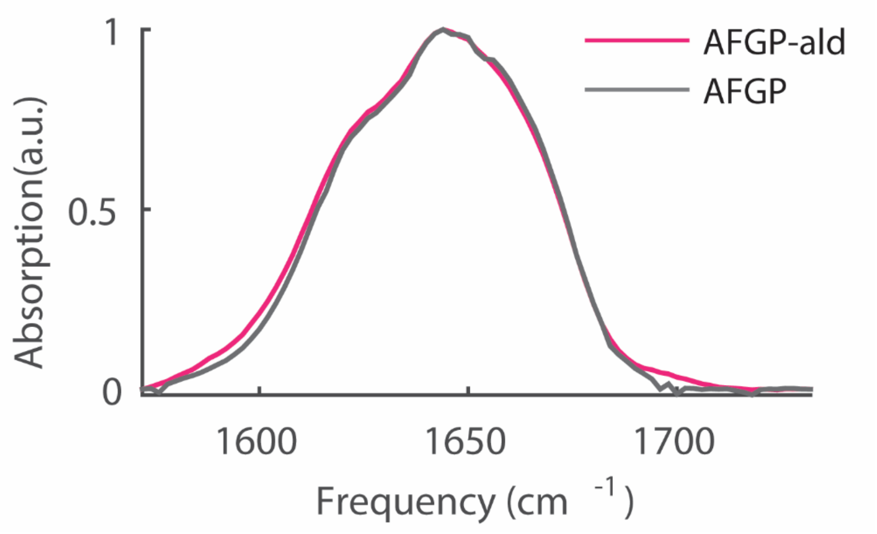

Figure S2. Linear IR spectra of AFGP and AFGP-ald. The new signal at ~1700 cm^{-1} can be assigned to the newly formed aldehyde group. Spectra were measured in heavy water and at a concentration of 2 wt%.

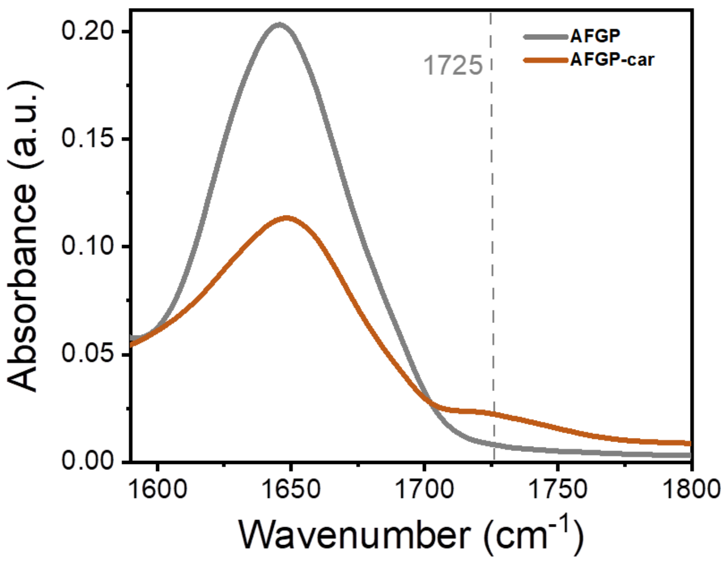

Figure S3. Linear IR spectra of AFGP and AFGP-car. The new signal at ~1730 cm^{-1} can be assigned to the newly formed carboxylic acid group. Spectra were taken from solid compounds.

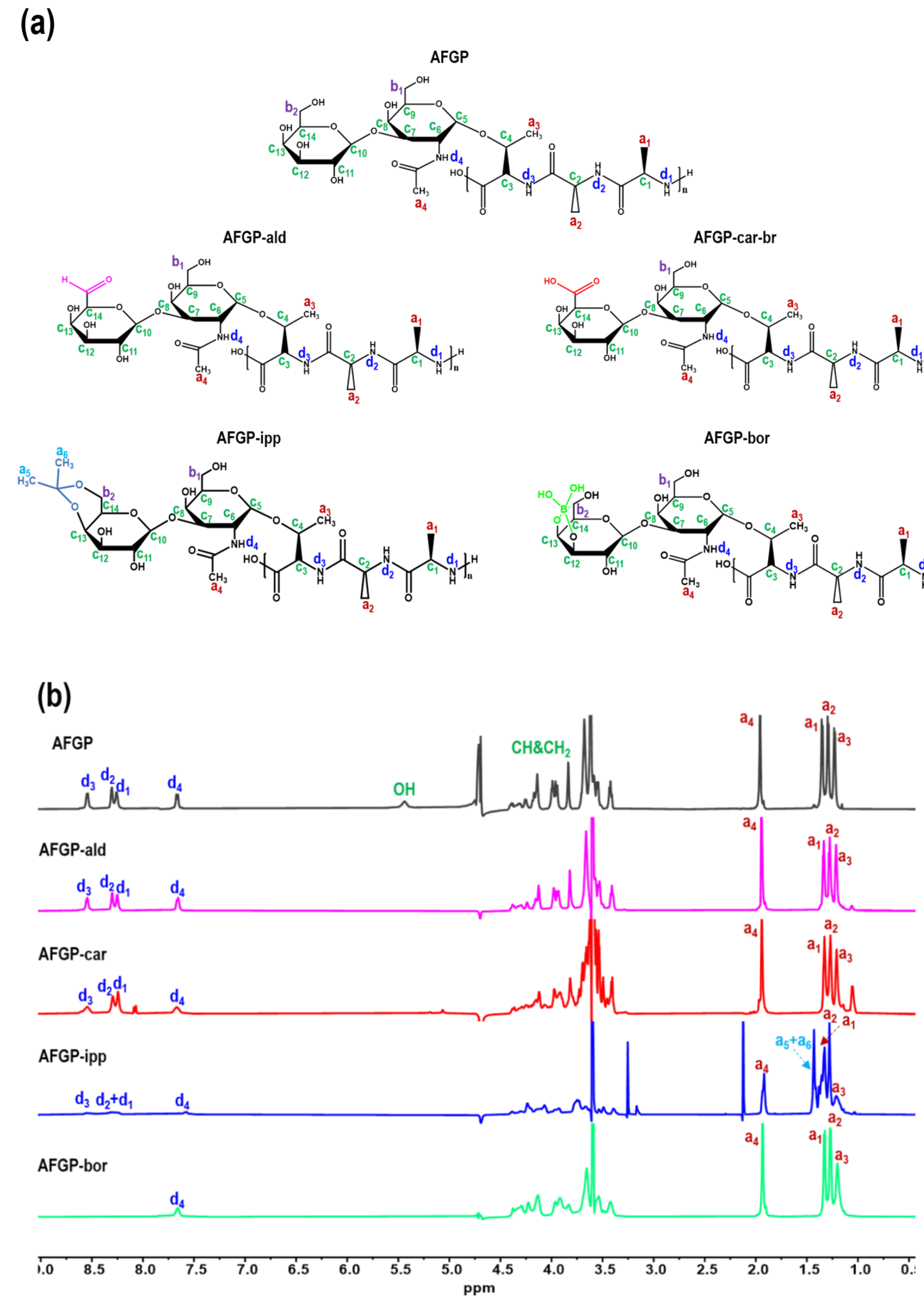
(a)
AFGP
AFGP-ald
AFGP-car-br
AFGP-ipp
AFGP-bor
(b)
AFGP
AFGP-ald
AFGP-car
AFGP-ipp
AFGP-bor
CH&CH2
OH
ppm

(c)

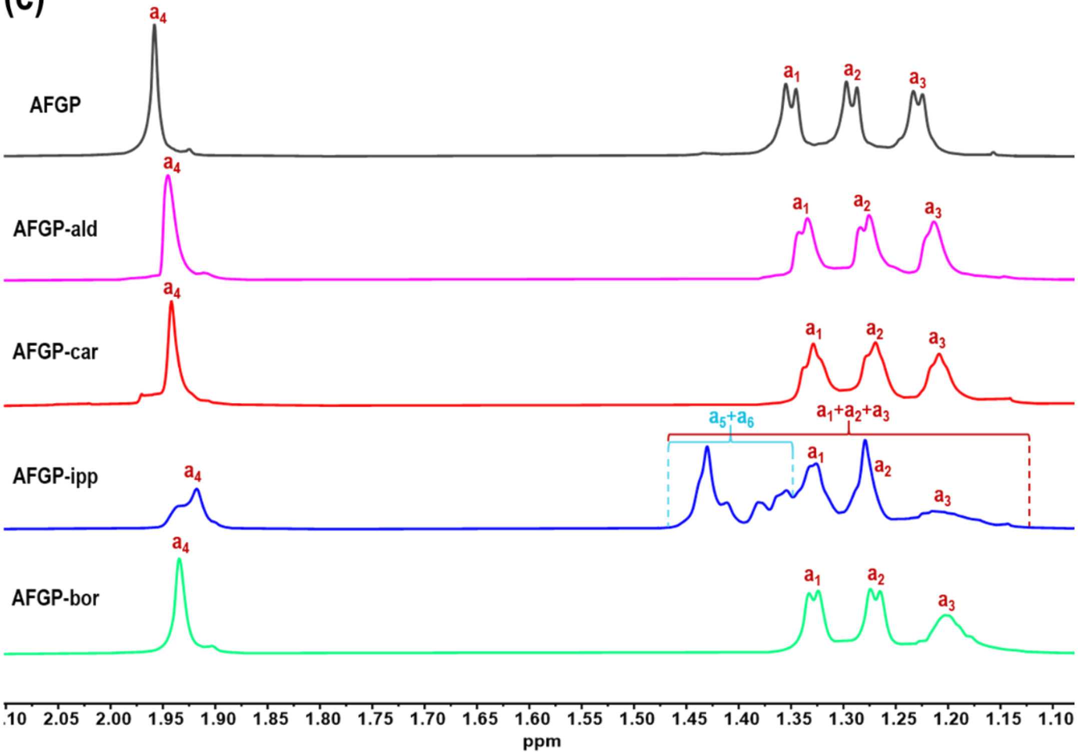
AFGP
AFGP-ald
AFGP-car
AFGP-ipp
AFGP-bor
a4
a1
a2
a3
a5+a6
a1+a2+a3
ppm

(d)

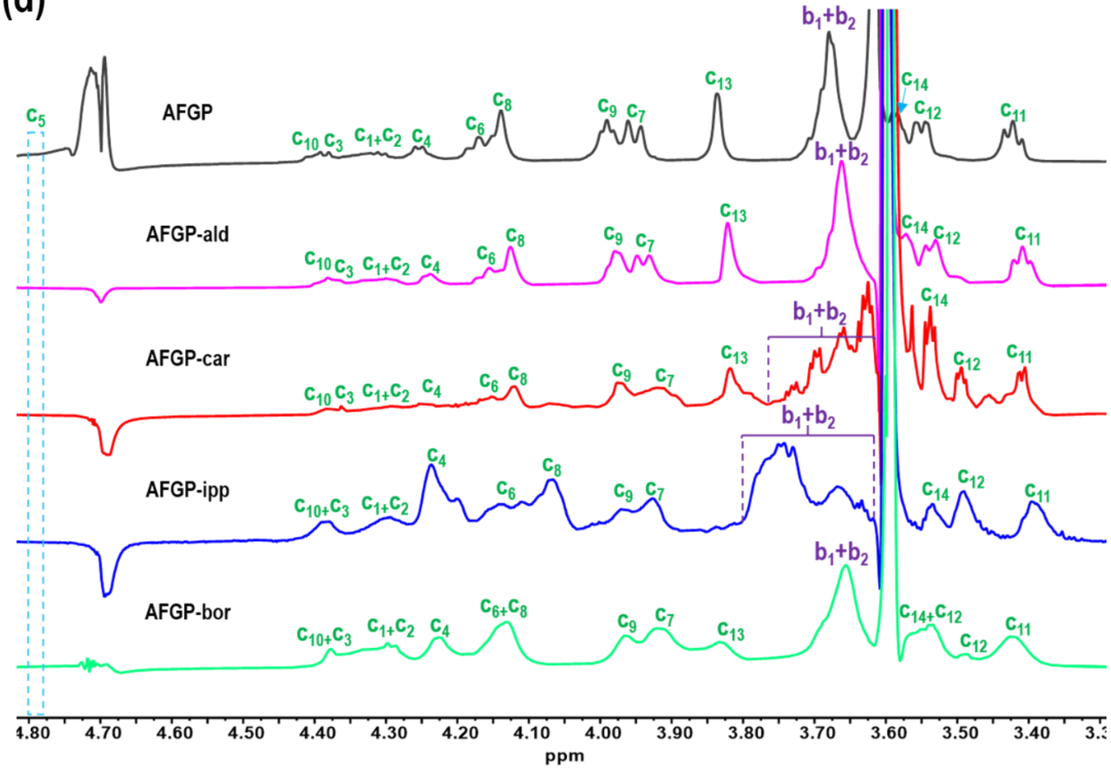
AFGP
AFGP-ald
AFGP-car
AFGP-ipp
AFGP-bor
c5
b1+b2
c10 c3
c1+c2
c4
c6
c8
c9
c7
c13
c14
c12
c11
c10+c3
c6+c8
c14+c12
ppm

Figure S4. Chemical structures and ^{1}H-NMR spectra of natural AFGP and the different variants measured in 10% D_2O/90% H_2O and at 700 MHz. (a) Chemical structures of natural AFGP and the variants. b-d) ^{1}H-NMR spectra of natural AFGP and the modified variants shown between (b) 1.0 ppm to 9.0 ppm, (c) 1 ppm to 2 ppm, (d) and 3.3 ppm to 4.5 ppm (c5 is assigned to ~4.79 ppm in the blue frame based on TOCSY measurements. For the AFGP variants, c5 can not be detected due to the water presaturation).

The NMR signal at ~5.4 ppm (Figure 2b) is an interplay of all exchangeable hydroxyl protons in the system and depends on all the exchange values between these protons in agreement with prior study[12-13]. The modifications of hydroxyl groups on the disaccharide of natural AFGPs can change the acidity and the exchange rate of remaining hydroxyl protons and all the other protons, which affects their position, intensity, and line width of the signal. The different exchange behavior may easily lead the remaining OH signal to overlap with the dominant water signal[12]. Therefore, the apparent disappearance of OH signal should be explained by the exchange of the hydroxyl groups with all the other protons affected by the modifications, which are also exchangeable like the water protons or all the other remaining acidic protons.

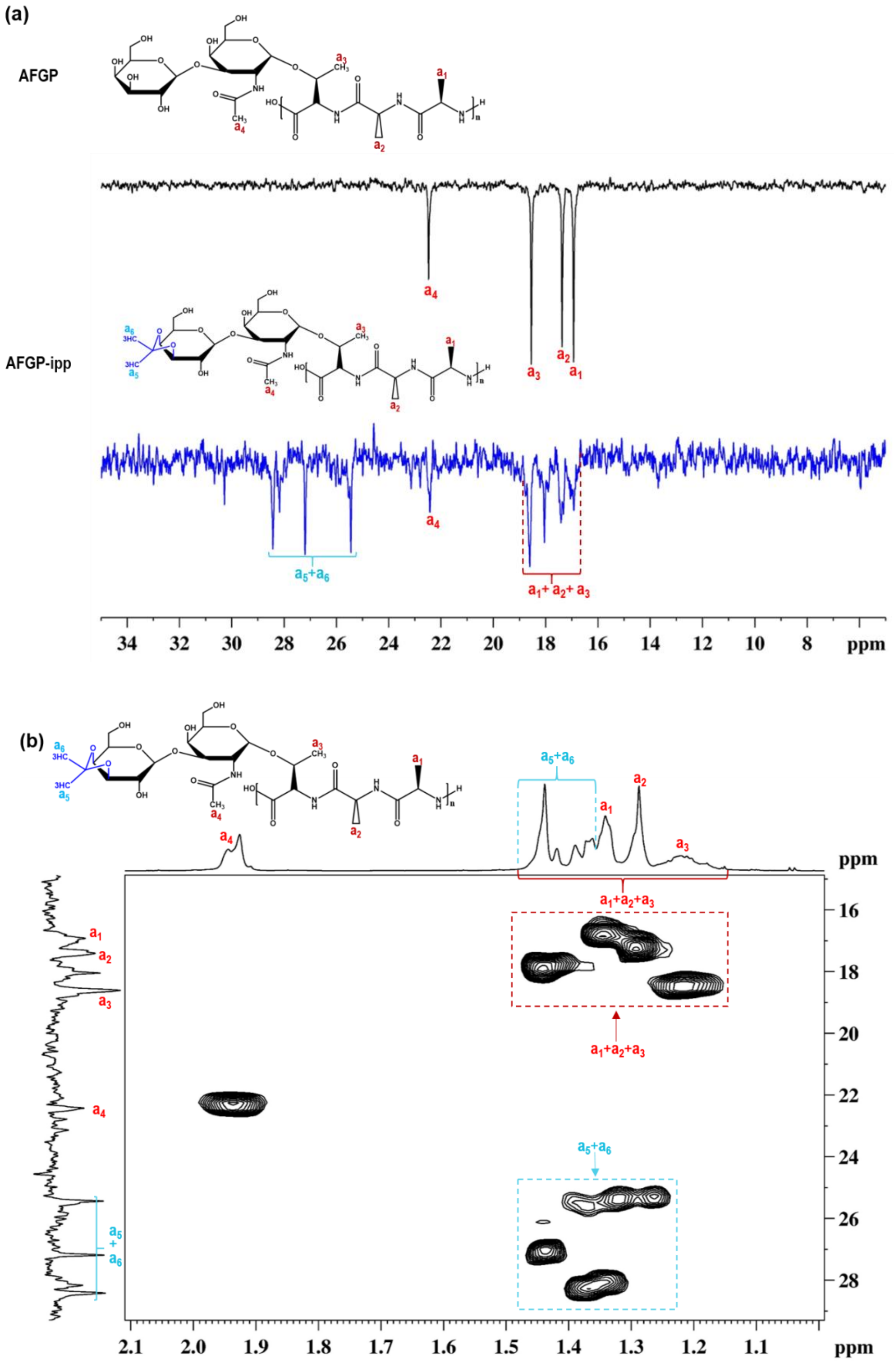

Figure S5. ^{13}C-NMR and 2D ^{1}H, ^{13}C-HSQC ^{1}J spectra of AFGP and AFGP-ipp. (a) ^{13}C-NMR spectra show new peaks at 25.4 and 27.1 ppm which are assigned to the isopropylidene group. (b) 2D ^{1}H, ^{13}C-HSQC ^{1}J

spectra show peaks at 18.0 ppm, 28.1 ppm, and 28.5 ppm in the ^{13}C-NMR which are also due to AFGP-ipp. The 2D HSQC spectra show additional signals ~1.2 ppm to ~1.5 ppm in the proton region which belong to the methyl groups from the isopropylidene and the corresponding carbon signals are observed ~25 to ~29 ppm.

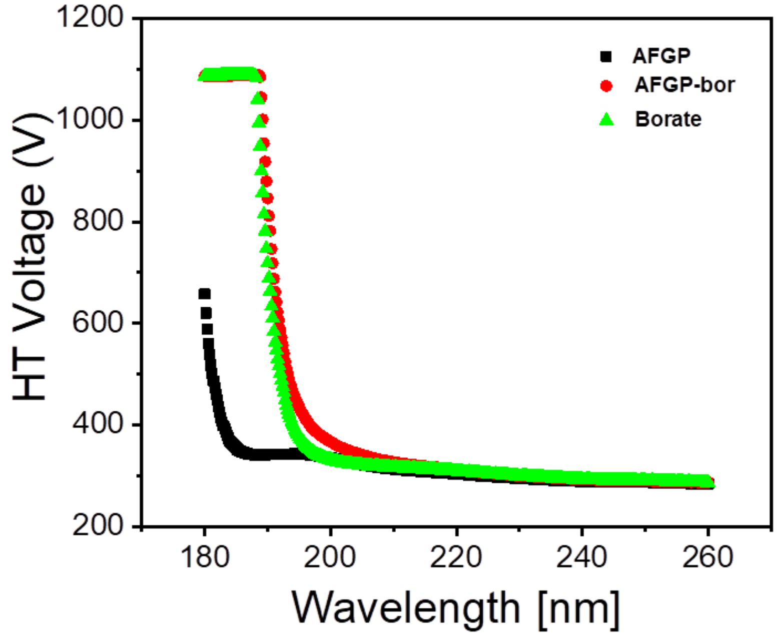

Figure S6. HT(V) spectrum of AFGP and AFGP-bor variant. The samples concentration is (0.1 mg/mL). The HT voltage of AFGP-bor and borate solution (0.3 M, pH=9) are above 600 that results in non-observable CD signals due to the overwhelming absorption effects.

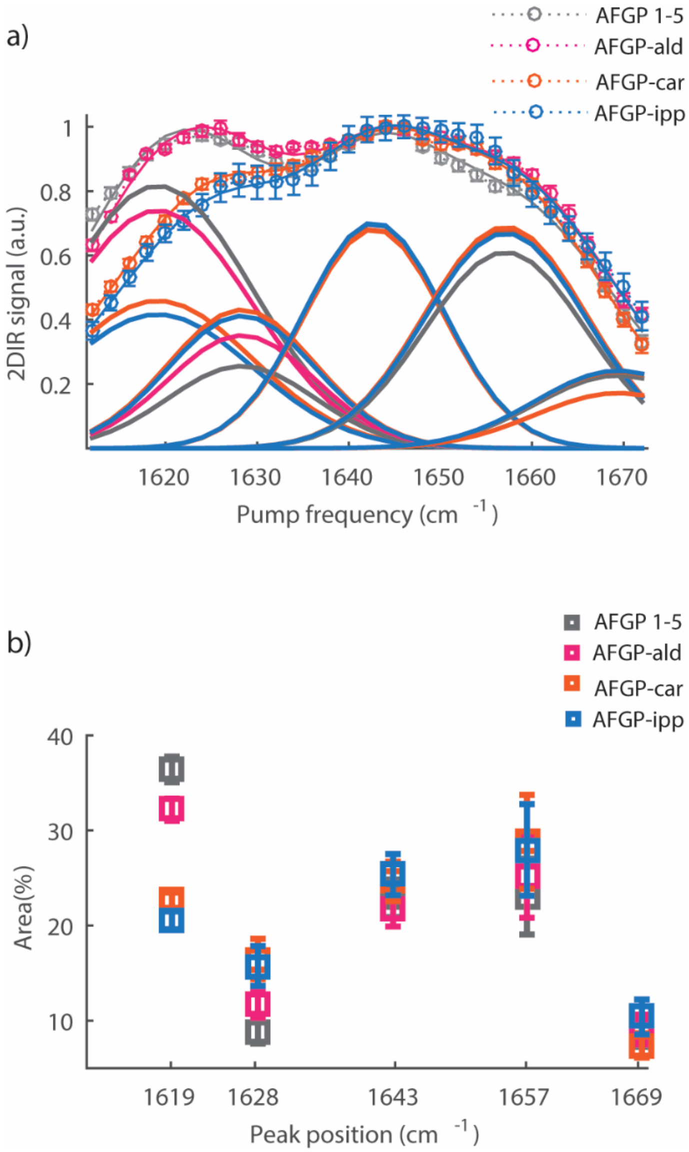

Figure S7. Anti-diagonal slices and normalized areas of the five 2D-IR bands for AFGP and the variants. (a) Anti-diagonal slices for solutions of natural AFGP and AFGP variants in heavy water and at a concentration of 2 wt% at 5°C. The raw data are represented by circles, and the lines represent the fits. The spectra were fitted with five Gaussian-shaped bands based on our previous study on AFGPs[14]. (b) Normalized areas of the five bands for the natural AFGP and AFGP variants.

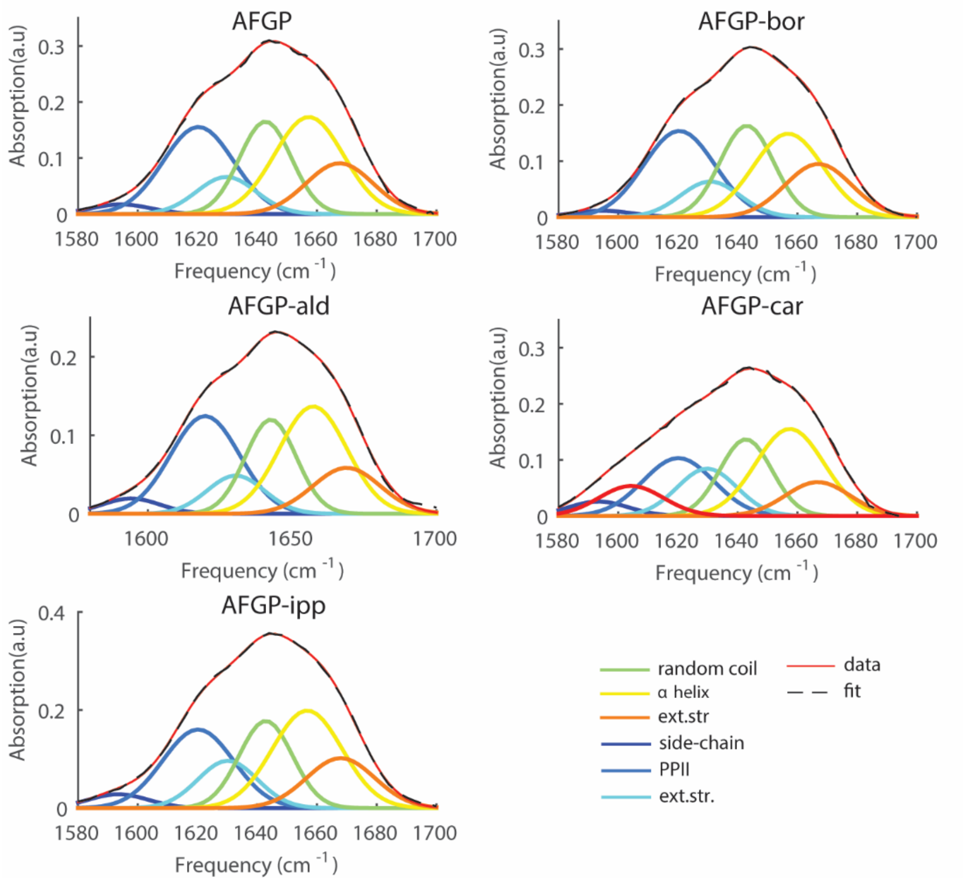

Figure S8. Linear IR spectra and fits for AFGP and variant solutions in heavy water and at a concentration of 2 wt%. The linear IR spectra were measured at a temperature of 5 °C. To fit the linear spectra we use 6 (7 in the case of AFGP-car) Gaussian-shaped bands. The widths and the centre frequencies of the bands are constrained to be within ±3 cm^{-1} from the values reported in our previous work on natural AFGPs [14]. In case of AFGP-car the centre frequency was found by studying the cross-peak between anti-symmetric and symmetric stretching vibrations of the carboxylate anion group. The width of this band was set to be as that of the anti-symmetric stretching vibration in a sample of glucuronic acid in heavy water at a concentration of 50 mM.

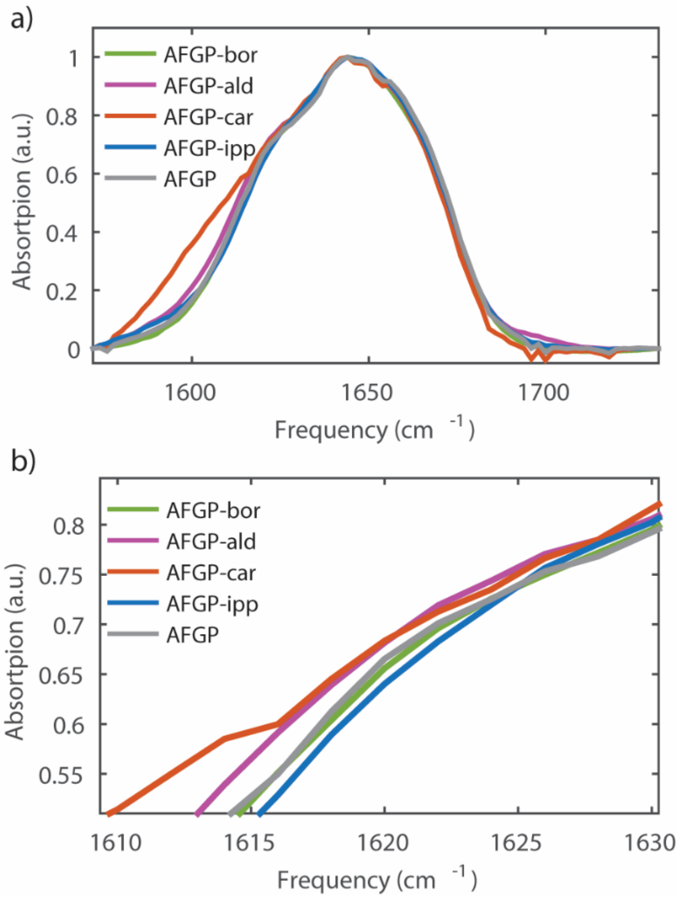

Figure S9. Linear IR spectra and detailed PPII absorption of AFGP and variants solutions. (a) Linear IR spectra of AFGP and variants solutions in heavy water at a concentration of 2 wt%. The linear IR spectra were measured at a temperature of 5 °C. (b) Detail of the absorption in the PPII vibrational region. The higher intensity in the AFGP-car is due to the presence of the absorption band of the anti-symmetric vibration of the carboxylate group at 1605 cm^{-1}.

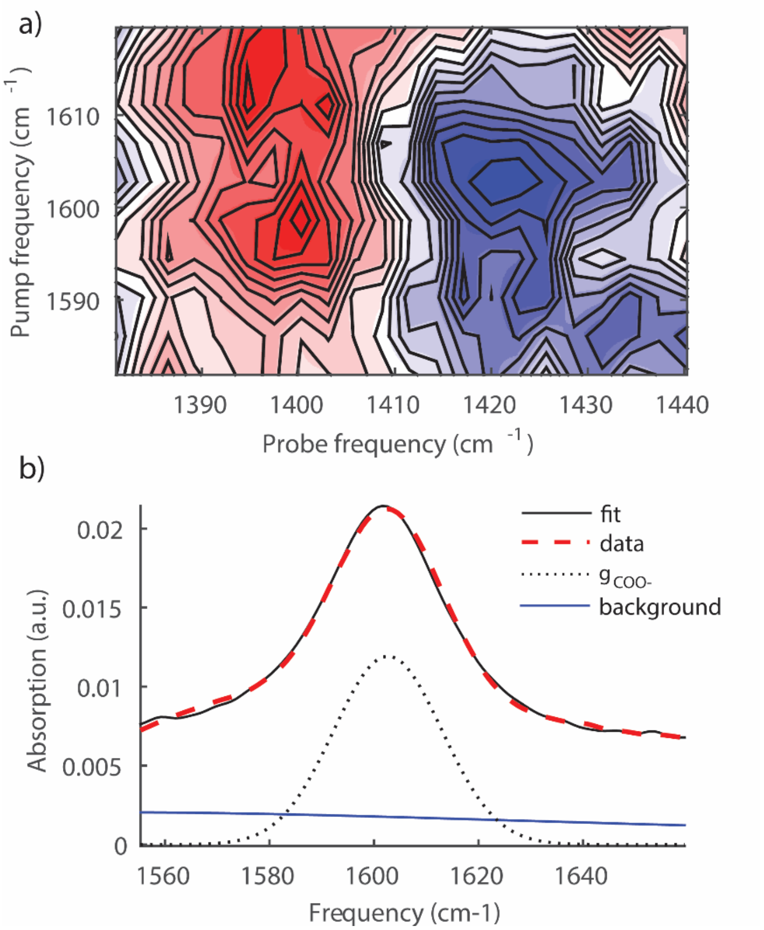

Figure S10. 2D-IR spectrum of AFGP-car and linear spectrum of glucuronic acid. (a) 2D-IR spectrum of AFGP-car at a concentration of 2 wt% at *Tw* of 0.5 ps. By exciting the anti-symmetric stretching vibration and probing the symmetric stretching of the carboxylate group, we are able to find the centre frequency of the anti-symmetric stretching vibration, which absorbs around 1605±3 cm^{-1}. (b) Linear spectrum of glucuronic acid in heavy water at a concentration of 55 mM. We observe a single band around 1605 cm^{-1}, which we fit by using a single Gaussian-shaped peak with a width of 34±3 cm^{-1}.

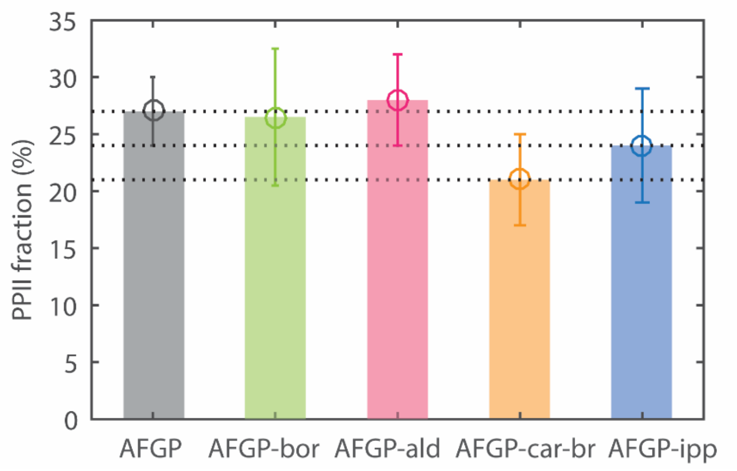

Figure S11. Relative areas of the PPII band for AFGP and variant solutions in heavy water at a concentration of 2 wt% and at a temperature of 5 °C. The areas were extracted from Figure S7.

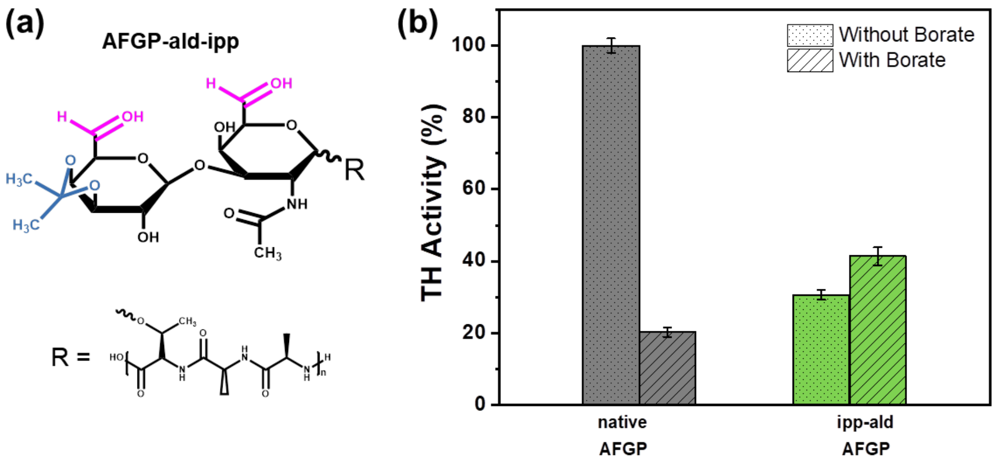

Figure S12. (a) Schematic representation of the modified variant AFGP-ipp-ald. (b) TH activity of AFGP and the modified variant AFGP-ipp-ald. Experiments were performed at least three times, and the error bars represent the standard deviation between the individual measurements.

Movie S1 (separate file). Growth habit of native $AFGP_{1-5}$ at 10 mg/mL in the hysteresis gap.

With the native AFGP it is difficult to melt the ice back to a round or oblong single crystal. In this video the single crystal has a protrusion at one end which morphs into the apex of the HBP as it is cooled. In other trials the seed is usually oblong in shape and as the temperature is decreased it grows into a blunt hexagonal bipyramid (BHBP), then morphs into a hexagonal bipyramid with a c-to-a ratio of 1.5-2 which is stable until the burst point. At the freezing point or burst point a fine spicule usually propagates from both ends to the water/oil interface followed by bundles of spicules parallel to the initial spicules. The spicules appear to thicken following their extension to the interface.

Movie S2 (separate file). Growth habit of AFGP-ald at 10 mg/mL in the hysteresis gap.

The growth morphology is the same or at least very similar to the native AFGP. In this video the cooling of the seed crystal was started from the HBP stage.

Movie S3 (separate file). Growth of AFGP-car at 10 mg/mL H_2O in the hysteresis gap. As the temperature is slowly lowered, the round seed crystal (10 µm diam) rapidly grows into a BHPB and then a sharp tipped HBP. It continues to grow in "fits and starts" until both tips reach the water/oil interface at which point a-axis growth becomes predominant.

Movie S4 (separate file). Growth habit of AFGP-ipp at 10 mg/mL in the hysteresis gap.

The round seed rapidly grows through the BHPB stage forming a sharp tipped HBP. It continues to grow in "fits and starts" until both tips reach the water/oil interface and a-axes growth becomes predominant. As growth continues without lowering the temperature the crystal grows laterally eventually forming one large crystal that replaces the liquid phase. A similar growth pattern is seen with the native AFGP in 0.3M sodium borate buffer at pH 9.

Movie S5 (separate file). Growth of AFGP 8 isoform at 10 mg/mL H_20 in the hysteresis gap.

The initial round crystal grew into a BHBP, morphed into a HBP which steadily grew as the temperature was lowered until the tips reached the interface and then a-axis lateral growth ensued without further temperature decrease.

SI References

1. Evans, C. W.; Gubala, V.; Nooney, R.; Williams, D. E.; Brimble, M. A.; Devries, A. L., How do Antarctic notothenioid fishes cope with internal ice? A novel function for antifreeze glycoproteins. *Antarctic Science* **2011,** *23* (1), 57-64.
2. Liu, M.; Mao, X.-a.; Ye, C.; Huang, H.; Nicholson, J. K.; Lindon, J. C., Improved WATERGATE Pulse Sequences for Solvent Suppression in NMR Spectroscopy. *Journal of Magnetic Resonance* **1998,** *132* (1), 125-129.
3. Palmer, A. G.; Cavanagh, J.; Wright, P. E.; Rance, M., Sensitivity improvement in proton-detected two-dimensional heteronuclear correlation NMR spectroscopy. *Journal of Magnetic Resonance (1969)* **1991,** *93* (1), 151-170.
4. Kay, L.; Keifer, P.; Saarinen, T., Pure absorption gradient enhanced heteronuclear single quantum correlation spectroscopy with improved sensitivity. *Journal of the American Chemical Society* **1992,** *114* (26), 10663-10665.
5. Schleucher, J.; Schwendinger, M.; Sattler, M.; Schmidt, P.; Schedletzky, O.; Glaser, S. J.; Sørensen, O. W.; Griesinger, C., A general enhancement scheme in heteronuclear multidimensional NMR employing pulsed field gradients. *Journal of biomolecular NMR* **1994,** *4* (2), 301-306.
6. Selig, O.; Siffels, R.; Rezus, Y. L. A., Ultrasensitive Ultrafast Vibrational Spectroscopy Employing the Near Field of Gold Nanoantennas. *Physical Review Letters* **2015,** *114* (23), 233004.
7. Meister, K.; Duman, J. G.; Xu, Y.; DeVries, A. L.; Leitner, D. M.; Havenith, M., The Role of Sulfates on Antifreeze Protein Activity. *The Journal of Physical Chemistry B* **2014,** *118* (28), 7920-7924.
8. Geng, H.; Liu, X.; Shi, G.; Bai, G.; Ma, J.; Chen, J.; Wu, Z.; Song, Y.; Fang, H.; Wang, J. J. A. C. I. E., Graphene Oxide Restricts Growth and Recrystallization of Ice Crystals. *Angewandte Chemie International Edition* **2017,** *56* (4), 997-1001.
9. Budke, C.; Dreyer, A.; Jaeger, J.; Gimpel, K.; Berkemeier, T.; Bonin, A. S.; Nagel, L.; Plattner, C.; DeVries, A. L.; Sewald, N.; Koop, T., Quantitative Efficacy Classification of Ice Recrystallization Inhibition Agents. *Crystal Growth & Design* **2014,** *14* (9), 4285-4294.
10. Budke, C.; Heggemann, C.; Koch, M.; Sewald, N.; Koop, T., Ice recrystallization kinetics in the presence of synthetic antifreeze glycoprotein analogues using the framework of LSW theory. *The Journal of Physical Chemistry B* **2009,** *113* (9), 2865-2873.
11. Olijve, L. L. C.; Oude Vrielink, A. S.; Voets, I. K., A Simple and Quantitative Method to Evaluate Ice Recrystallization Kinetics Using the Circle Hough Transform Algorithm. *Crystal Growth & Design* **2016,** *16* (8), 4190-4195.
12. Lane, A. N.; Hays, L. M.; Tsvetkova, N.; Feeney, R. E.; Crowe, L. M.; Crowe, J. H., Comparison of the solution conformation and dynamics of antifreeze glycoproteins from Antarctic fish. *Biophysical Journal* **2000,** *78* (6), 3195-3207.

13. Lane, A. N.; Hays, L. M.; Crowe, L. M.; Crowe, J. H.; Feeney, R. E., Conformational and dynamic properties of a 14 residue antifreeze glycopeptide from Antarctic cod. *Protein Science* **1998,** *7* (7), 1555-1563.
14. Giubertoni, G.; Meister, K.; DeVries, A. L.; Bakker, H. J., Determination of the Solution Structure of Antifreeze Glycoproteins Using Two-Dimensional Infrared Spectroscopy. *The journal of physical chemistry letters* **2019,** *10* (3), 352-357.
